# The Need for a Person-Oriented Approach in Research and Therapy: Treatment Personalization in Clinical Psychology and Psychotherapy

**DOI:** 10.3390/jcm15114361

**Published:** 2026-06-04

**Authors:** Dariusz Krok

**Affiliations:** Institute of Psychology, University of Opole, 45-040 Opole, Poland; dkrok@uni.opole.pl; Tel.: +48-77-452-73-70

## 1. Introduction

Throughout the modern development of clinical psychology and psychotherapy, one of the major challenges has been understanding individual patients and their mental needs to comprehend how to tailor treatment most effectively to their specific needs. Every mental or physical illness often triggers in patients a tendency to search for and explain the causes of their illness in terms of psychological characteristics (e.g., illness perceptions, stress vulnerability or emotional antecedents) as well as lifestyle and environmental risk factors (e.g., harmful lifestyle, chemical contamination or lack of a proper diet) [1,2]. The underlying motive for this process is a need for understanding the causes and consequences of the illness, which has a clear impact on a personalized approach to treatment.

In clinical psychology and practice, given the wide range of theoretical models and effective interventions available for each mental health diagnosis, clinicians are required to make multiple decisions in order to tailor theory and psychotherapy to the needs of individual patients. As a consequence, personalization has traditionally involved therapists selecting interventions based on assessment and case formulation, deciding in real time which techniques to apply during therapy, relying on theoretical orientations, or drawing upon professional experience and judgment [3,4]. Therapists tend to choose a specific therapeutic model or treatment manual from several options to incorporate the patient’s personal characteristics into treatment. Clinicians also determine when to modify the treatment approach, for instance, by adjusting strategies to the patient’s personality traits or social conditions, as well as determining how often sessions should be scheduled and when therapy should be concluded. This approach highlights the key decision points involved in delivering psychotherapy and regulates evidence-based guidelines to support these clinical choices.

The person-oriented approach is an increasingly important direction in contemporary psychological research and clinical practice. This development is linked to the urgent need to take into account specific inter-individual differences, address each person’s unique needs, and adapt therapy to cultural contexts [5,6]. In contrast to the traditional variable-oriented approach, which focuses on analyzing individual variables and the relationships between them at the population level, the person-oriented approach assumes that the individual is a dynamic and integrated system of traits, experiences, and psychological mechanisms. Every person is psychologically unique and distinctive, which means that human functioning cannot be fully explained through the analysis of isolated factors but instead requires consideration of their interrelated configurations and individual patterns of functioning.

In psychological research and practice, the need to use a person-oriented approach arises primarily from the high heterogeneity of psychopathological phenomena and individual differences in the course of mental problems, coping strategies, and responses to treatment. Individuals who meet the same diagnostic criteria may differ significantly in terms of symptom severity, level of psychological distress, personality structure, environmental factors, and adaptive mechanisms [7]. Consequently, traditional models based on group mean analyses may overlook this complexity, leading to oversimplification and limited clinical validity. In contrast, the person-oriented approach enables the identification of relatively homogeneous subgroups of patients characterized by distinct profiles of psychological functioning. This can play a particularly important role in the analysis of such phenomena as stress coping, illness perception, emotion regulation, anxiety behaviors, self-esteem, and personality disorders [5,8,9].

Through an individualized approach in clinical psychology and psychotherapy, it becomes possible to more effectively identify specific configurations of risk and protective factors associated with the development of mental disorders, as well as to more accurately select and apply therapeutic methods. This also allows for more precise prediction of the course of illness, the level of psychosocial functioning, and the effectiveness of particular forms of therapy. When facing psychological challenges, individuals encounter a wide range of stressors of varying intensity, which activate characteristic and individualized coping strategies as well as efforts to seek appropriate psychological support. Contemporary treatment models increasingly emphasize the need to personalize such therapeutic interventions and adapt methods to the individual needs of the patient [6,10]. This tactic enables consideration of the unique configuration of symptoms, emotional experiences, attachment styles, coping strategies, and beliefs about oneself and the world. As a result, it increases the potential for designing more appropriate and effective therapeutic interventions.

Although such an individualized approach appears intuitively valuable, there are ongoing substantive and intensive debates in contemporary clinical psychology concerning the role of individual factors, attempts to improve therapeutic outcomes, and theory-driven approaches to personalization. Personalization in research aims at the most precise possible identification of individual factors, the delineation of inter-individual differences, and the specification of relevant developmental determinants [5,6]. In addition, personalization in treatment may include dimensions such as determining the appropriate type of psychological and medical care, selecting optimal therapeutic strategies, identifying key intervention targets, or matching patients with specific therapists [3,7]. Advances in statistical analyses have also significantly contributed to identifying factors and mechanisms relevant to understanding individual determinants and developing potential personalized interventions [11]. This has expanded the scope of clinical psychological research and improved therapeutic decision-making.

## 2. The Substantive Analysis of Published Articles

The aim of this Special Issue was to identify and investigate individual patient characteristics and the main psychological and psychotherapeutic ways directed at optimizing treatment. The theoretical foundations, empirical findings, and practical applications for personalized treatment in clinical psychology and psychotherapy were examined. The following articles interestingly analyzed previous findings and theoretical approaches, broadening substantially the scope of our knowledge.

The first article, written by Konieczny et al. (contribution 1), presented a Polish adaptation and psychometric validation of the self-report questionnaire developed to detect meteoropathy and meteorosensitivity (METEO-Q) in three different samples: healthy, cardiac, and psychiatric patients. A sample of 1128 adults was used, including healthy individuals, cardiac patients, and people with mental disorders. The researchers evaluated the questionnaire’s structure, reliability, and validity using factor analyses and comparisons across groups. The main results demonstrated that a two-factor model (i.e., meteoropathy and meteosensitivity) fit the data better than a single-factor model, consistent with findings from other countries. The scale showed high internal consistency and worked similarly across clinical groups; for instance, people with mental disorders scored highest, while cardiac patients scored lower than healthy participants.

The next article written by Krok et al. (contribution 2) investigated the relationships among illness perception, fear of recurrence, and generalized anxiety in post-treatment thoracic cancer patients within a serial multiple model, including also meaning-making and changes in beliefs and goals. Examining 284 post-treatment thoracic cancer patients and using regression and mediation analyses, researchers examined both direct and indirect relationships. Results showed that negative illness perceptions were linked to greater fear of recurrence and anxiety, while positive illness perceptions were associated with lower distress. In addition, meaning-making and changes in beliefs and goals functioned as mediators. The findings suggest that psychological adjustment after cancer is influenced not only by how patients perceive their illness but also by how they create meaning and adapt their personal beliefs and goals.

Dymecka et al.’s study (contribution 3) focused on how pandemic-related stress, sense of coherence, and social support were associated with fear of childbirth among pregnant women during the COVID-19 pandemic. The study included 232 pregnant women, and its results showed that higher pandemic-related stress—especially stress related to infection and preparedness for childbirth—was linked to greater fear of childbirth. In contrast, a stronger sense of coherence was associated with lower fear of childbirth, while social support showed only weak associations. Regression analyses indicated that preparedness stress and sense of coherence were the main predictors of fear of childbirth.

The fourth article by Furmańska et al. (contribution 4) investigated the relationships between loneliness, self-esteem, and phubbing behavior (ignoring others in favor of using a smartphone). A group of 201 adults aged 18–75 completed questionnaires measuring loneliness, self-esteem, and phubbing, along with phone and social media use habits. Results showed that higher loneliness was associated with more frequent phubbing and lower self-esteem. In turn, lower self-esteem was related to greater phubbing behavior. The main finding demonstrated that self-esteem mediated the relationship between loneliness and phubbing.

The next article (Solska et al.; contribution 5) aimed to explore the relationship between perfectionism and orthorexic behaviors (i.e., an unhealthy obsession with eating only “healthy” or “clean” foods) and examined whether self-compassion mediates this relationship. Based on a research group of 227 adults aged 18–55, the results showed that maladaptive perfectionism was positively associated with orthorexic behaviors. Both adaptive and maladaptive perfectionism were also related to different aspects of self-compassion. Importantly, self-compassion was found to mediate the relationship between perfectionism and orthorexic tendencies, suggesting that lower self-compassion may help explain why perfectionistic individuals are more vulnerable to obsessive healthy-eating behaviors.

The next study by Dymecka et al. (contribution 6) was to examine the associations between fear of COVID-19, pandemic-related stress, fear of childbirth, hardiness, and life satisfaction among pregnant women during the COVID-19 pandemic. A sample of 247 pregnant women aged 18–39 was assessed. Results showed that fear of COVID-19 was strongly associated with higher infection-related stress, preparedness stress, and fear of childbirth. In contrast, hardiness and life satisfaction were negatively associated with fear of childbirth, meaning that women with greater psychological resilience and higher life satisfaction experienced less childbirth-related fear. This indicates that strengthening personal psychological resources, especially hardiness, may help women cope more effectively during pandemics and other crisis situations.

Zarzycka et al.’s study (contribution 7) evaluated the preliminary effectiveness of a six-day outpatient psychotherapeutic training based on the Duc in Altum Therapy (DIAT) approach in reducing anxiety, depression, and stress in adults. The intervention involved 109 participants who took part in intensive supportive and experiential group therapy over six days. Results showed significant reductions in anxiety, depression, and stress following the training, and these improvements were maintained at the three-month follow-up assessment. Although the study did not include a control group, limiting conclusions about causality, the findings provide preliminary evidence that DIAT may be an effective brief psychotherapeutic intervention for improving mental health and reducing emotional distress.

The eighth article (Sołtys and Wnuk, contribution 8) aimed to assess psychological factors influencing the involvement of caregivers of individuals with Alzheimer’s disease, focusing on stress, sense of coherence, and time spent caregiving. Drawing on a sample of 100 caregivers of individuals with Alzheimer’s disease, the study tested a moderated mediation model in which stress influences caregiver involvement indirectly through a sense of coherence, while the strength of this relationship depends on caregiving time. The findings revealed that sense of coherence mediated the relationship between stress and caregiving involvement, and this effect became stronger when caregivers spent more time providing care. This implies that strengthening resilience factors may be beneficial in interventions aimed at improving caregiver mental health and preventing burnout.

The next study by Konieczny et al. (contribution 9) validated the Polish version of the Somatosensory Amplification Scale (SSAS-PL) and evaluated its psychometric properties in both healthy individuals and clinical populations. Results showed that the SSAS-PL has good internal consistency and a stable one-factor structure after removing one item. The scale demonstrated measurement invariance across groups, as well as good convergent validity with somatic symptoms and health anxiety, and appropriate discriminant validity from broader psychopathology measures. Overall, the SSAS-PL is a reliable and valid tool for assessing somatosensory amplification in Polish populations, suitable for both research and clinical use.

Next, Riedl et al. (contribution 10) investigated how loneliness, depressive symptoms, and mentalizing abilities are related to pain severity in patients with rheumatic diseases. Using structural equation modeling, the researchers tested whether mentalizing helps explain the relationship between loneliness and pain severity. Loneliness turned out to be associated with greater pain severity, but this relationship was fully explained by a sequential pathway involving reduced mentalizing and increased pain-related depressive symptoms. This indicates that enhancing mentalizing abilities can serve as a protective factor and may help improve outcomes in chronic pain patients.

In the eleventh study, Kasprzak et al. (contribution 11) examined the relationship between perceived hope, dispositional hope, and various aspects of psychological and physical well-being, as well as validated a Polish version of the Perceived Hope Scale (PHS-PL). Using data from 1608 adults in an international online survey, the researchers compared how both forms of hope relate to mental and physical health outcomes. The findings demonstrated that perceived hope was more strongly associated with psychological well-being than dispositional hope, while both were similarly related to physical health. Hope therefore appears to be an important health-promoting psychological resource that is a particularly useful construct for assessing well-being across different populations.

The next article (Larionow et al.; contribution 12) investigated whether alexithymia was a stable personality trait or a temporary state linked to psychological distress by assessing its stability over seven months. Statistical analyses revealed that alexithymia levels remained stable over time, with no significant changes across measurements. In contrast, other mental health variables did fluctuate. Overall, the results support the view that alexithymia functions as a stable trait rather than a short-term reaction to distress, reinforcing its role as a risk factor for psychopathology.

Jung et al. (contribution 13) evaluated the effectiveness of digital cognitive behavioral therapy (dCBT) for panic disorder and agoraphobia and investigated whether specific therapeutic components influence its outcomes. A meta-analysis of 31 randomized controlled trials found that dCBT was moderately effective compared with passive controls but showed no advantage over traditional face-to-face CBT. This implies that differences in the content and structure of dCBT programs help explain variations in their effectiveness. In addition, incorporating key therapeutic components may enhance the efficacy of digital CBT interventions for panic disorder and agoraphobia.

The following study by Larionow et al. (contribution 14) validated the Polish version of the Emotion Regulation Questionnaire (ERQ) and examined patterns of emotion regulation strategies and their associations with mental health. Using a sample of 1197 Polish adults, the researchers confirmed a stable two-factor structure measuring cognitive reappraisal and expressive suppression, which was consistent across demographic groups and levels of psychological symptoms. They also identified four emotion regulation profiles: mainly reappraisal, mainly suppression, generally low regulation, and generally high regulation. The findings support theories of emotion regulation by showing that different strategy patterns are linked to distinct psychological outcomes.

The fifteenth article by Seok et al. (contribution 15) was to assess the effectiveness of Emotional Freedom Techniques (EFT) in reducing depressive symptoms. Based on 18 randomized controlled trials, the results showed that EFT had a large overall effect in reducing depression. The analysis also found that effectiveness varied depending on intervention characteristics: group-based EFT was more effective than individual formats, people with moderate depression benefited the most, and shorter interventions tended to produce strong results. EFT can thus be an effective intervention for depressive symptoms, particularly in structured group settings and for individuals with moderate severity.

Finally, Krok et al. (contribution 16) explored how attachment styles are related to illness acceptance through the mediational role of sense of coherence in couples coping with breast cancer. Using a dyadic approach, the study examined how each partner’s psychological characteristics affected both themselves and each other. Results showed that secure attachment and lower insecure attachment were linked to a stronger sense of coherence and greater illness acceptance in both patients and partners. More interestingly, a sense of coherence mediated most of the relationships between attachment styles and illness acceptance, both within individuals and between partners. This indicates that couples cope with breast cancer in an interconnected way, where both partners’ emotional and cognitive resources influence adjustment to illness.

In total, the current Special Issue comprises sixteen papers that met the methodological requirements and were finally accepted for publication. Their authors and titles along with bibliometric details are presented at the end of this article.

## 3. Conclusions and Future Research Implications

This Special Issue was designed to investigate and better understand the complex factors and dynamics associated with treatment personalization in clinical psychology and psychotherapy. In particular, it focused on examining a wide array of psychological factors and mechanisms related to coping with stress, emotion regulation, illness acceptance, depressive symptoms, self-esteem, and panic disorder. The Special Issue also considered how these elements contribute to clinical psychology and therapeutic methods. Different clinical groups were chosen as the focus population to ensure a representativeness and specific psychological mechanisms [5,11]. This approach appears particularly suitable for systematic and comparative research on the factors and mechanisms responsible for treatment personalization in clinical psychology and psychotherapy.

The findings of the studies included in this Special Issue convincingly demonstrate the important role of individual factors in psychological research and practice. This is particularly significant in the case of disorders with complex and multifactorial etiology, such as the psychological consequences of chronic illness [12], psychological difficulties related to pregnancy and childbirth [13], depression [14], or anxiety disorders [15], where traditional diagnostic categories are often insufficient to capture the individual’s true clinical picture. The findings showed that specific individual factors play a fundamental role in both physical and psychological functioning and are also involved in a range of mediating and moderating relationships [16]. They highlight the importance of examining how personal resources and vulnerabilities operate within broader biopsychosocial systems. Such an approach contributes to a more nuanced understanding of individual differences and supports the development of personalized interventions aimed at improving mental health, adaptation to illness, and overall quality of life. This also confirms that a personalized approach aligns with the development of contemporary clinical psychology and psychiatry, which aims to integrate biological, psychological, and social data in order to achieve a better understanding of individual functioning [4,6].

Future research should continue advancing personalized approaches in clinical psychology and psychotherapy by examining how personality and social differences influence psychological functioning and treatment outcomes as well as investigating more complex, mediational mechanisms underlying these differences. In addition, longitudinal and multimethod studies are needed to better understand the dynamic interactions among psychological resources, coping patterns, cognitive processes and emotional functioning across different stages of life and health conditions. Another important direction involves identifying distinct psychological profiles and person-centered analytic methods, which may explain heterogeneity in mental health outcomes and support individualized diagnosis and intervention planning. This is likely to ensure that person-oriented approaches remain important to particular individuals and applicable across different healthcare settings [4,7,12].

In conclusion, the studies presented in this Special Issue are consistent with the current trend in person-oriented research and the need for a more individualized understanding of human functioning in both research and clinical practice. This approach enables the identification of complex patterns of psychological functioning, a better explanation of individual differences, and the development of more accurate and effective forms of diagnosis and therapy. Accordingly, it represents an important direction in the development of contemporary clinical psychology and psychiatry.
